# Mood symptoms in cervical dystonia: Relationship with motor symptoms and quality of life

**DOI:** 10.1016/j.prdoa.2023.100186

**Published:** 2023-01-31

**Authors:** Shameer Rafee, Mahmood Al-Hinai, Gillian Douglas, Ihedinachi Ndukwe, Michael Hutchinson

**Affiliations:** a Department of Neurology, St Vincent’s University Hospital, Merrion Road, Dublin, Ireland; b School of Medicine, University College Dublin, Belfield, Dublin, Ireland

**Keywords:** Cervical dystonia, Mood disorder, Rating scales, Motor severity, Quality of life

## Abstract

**Background:**

Cervical dystonia (CD) has a high prevalence of anxiety and depression. The relationship between motor severity, mood symptoms and QoL is unclear and how to adequately assess these is also unknown. Instruments like the BAI, BDI and HADS are often used but items within these relating to somatic symptoms might influence the results.

**Methods:**

Patients with idiopathic cervical dystonia (CD) were included. The BAI, BDI, HADS, CIDP58 and TWSTRS2- severity score were used for assessment of motor, mood and QoL symptoms. Pearson’s correlations between motor and non-motor symptom scores were assessed. The psychometric properties of the psychiatric tools were measured and principal component analysis performed after identifying items that could correspond to somatic symptoms.

**Results:**

201 participants were included. 42% of participants had either significant depression or anxiety symptoms or both when measured by BAI and BDI and 51% of patients met criteria on HADS. HADS-A and HADS-D, BAI and BDI were poorly correlated with TWSTRS2-S. The HADS-A and HADS-D both showed strong correlation with the sleep subdomain of CDIP58. Psychometric and principal component analysis on 149/201 participants did not reveal factor loadings consistent with the a priori somatic groupings. However mean scores were higher for somatic items.

**Conclusion:**

A good score on the CDIP58, a commonly used tool, does not indicate mild disease severity or minimal mood symptoms. Minimal motor symptoms, similarly, also does not imply a positive QoL. Clinicians should be mindful on ideal methods for performing a holistic assessment of CD patients. This likely warrants a combination of motor, QoL and mood assessment tools.

## 1 Introduction

In cervical dystonia (CD), the prevalence of anxiety and depression is as high as 42 % [1], with lower frequencies seen in other focal dystonias [2]. The psychiatric features are well established and form a major component of the *non-motor* syndrome [2], [3], [4]; they are also the major driver of poor quality of life (QoL) in this disorder [5]. Recently it was proposed that psychiatric disorders in dystonia may be intrinsically linked and genetically driven [6]. The interplay between mood symptoms, quality of life (QoL) and motor features can be complex, and the ideal method for assessing these in clinical practice has not been established. Importantly, any instrument tracking disease severity should also measure changes in mood.

The Movement Disorders Task Force has recommended the Cervical Dystonia Impact Profile-58 (CDIP-58) and Toronto Western Spasmodic Torticollis Rating Scale-2 (TWSTRS2) as rating scales for CD [7]. The CDIP-58 provides a composite score of motor, quality of life and non-motor features [8]. It is a long, complex self-report with the advantage of including psychosocial factors. The TWSTRS2 is an objective method for scoring disease severity [9] but may be too long for routine clinical implementation.

No guideline exists for identifying and measuring psychiatric features- it is unclear if the CDIP-58 or TWSTRS2 can reliably detect presence of anxiety or depression. Several validated instruments like the Hospital Anxiety and Depression Scale (HADS) [10], Beck Anxiety Inventory (BAI) [11], Beck Depression Index-II (BDI-II) [12] have been frequently used [4], [13], [14]. These tools, however, were designed for psychiatric populations and may not be suitable for capturing mood symptoms in neurology patients. Also in CD, motor severity can confound the presence of *true* anxiety and depressive symptoms. Although it may be reasonable to suggest that worse motor severity results in excess mood symptoms, the evidence of a correlation between mood scores and motor symptoms has been conflicting [4], [15].

When using the BAI, BDI or HADS, it is possible that, even without an overt link, items contained within these scales referring to motor features (*somatic symptoms*), have a disproportionate impact on scores. Statements like “hands trembling” in BAI may artificially inflate the measure of anxiety or depression. Furthermore, the association between these measures and the CDIP-58 has not been assessed in any detail. Specifically, it would be prudent to determine whether certain domains within this QoL measure can act as a reliable indicator of excess mood symptoms.

Our objectives in this study were to:

1. Assess the correlations between motor severity (TWSTRS2), QoL (CDIP58) and mood scores.
2. Examine the psychometric properties of the BAI, BDI-II and HADS and evaluate the contribution of specific items to overall results in patients with CD.
3. Identify how somatic and non-somatic groupings affect mood scores.

## 2 Participants & methods

### 2.1 Participants

Patients with adult-onset idiopathic cervical dystonia (CD), attending for 3-monthly botulinum toxin (BoNT) treatment, were included in this study. These patients had extensive non-motor (psychiatric and quality of life) and motor symptom assessments using validated rating scales. All participants had provided informed consent. Participants were on stable BoNT dosing (<10 % variation in total dose in the preceding 12 months). Testing was performed at week 0 of the 3-monthly BoNT injection cycle to mitigate impact of treatment on outcomes.

This study was approved by the St Vincent’s University Hospital Research and Ethics Committee.

### 2.2 Study instruments

The following instruments for evaluating mood, disease severity and quality of life were employed:

1. **Beck Anxiety Inventory (BAI):** validated anxiety assessment measure. Score of ≥ 13 was used to indicate presence of significant anxiety symptoms [16].
2. **Beck Depression Index (BDI-II):** validated depression assessment measure. Score ≥ 13 was used to indicate presence of significant depression symptoms [11].
3. **Hospital Anxiety and Depression Scales (HADS-A, HADS-D):** validated anxiety and depression tools. A cut-off score of ≥ 8 was used to indicate presence of significant mood symptoms [10].
4. **Cervical Dystonia Impact Profile-58 (CDIP-58):** a commonly used, disease specific tool for studying of quality of life [8]. The CDIP-58 encompasses eight subscales measuring head and neck related symptoms, pain, walking, upper limb, sleep, annoyance, mood and psychosocial functioning. The relationship of specific subscales to mood symptoms and motor severity was probed. Higher scores indicate more significant impairment of function (maximum score of 100).
5. **Toronto Western Spasmodic Torticollis Rating Scale 2 (TWSTRS2):** extensively validated tool used in cervical dystonia research for measuring dystonia severity, disability, pain and psychology [17]. Only TWSTRS2 severity scale (TWSTRS2-S,measuring the severity of motor features) was used for this study.

First a descriptive analysis was performed of our cohort. Next we sought to review the interaction between mood rating scales and QoL and severity measures. The BAI, BDI-II, HADS-A, HADS-D were compared to the CDIP-58 and TWSTRS2 using independent samples *t*-test to compare mean differences. Correlations were assessed using Pearson’s *r* as data was mostly normally distributed (poor correlation r < 0.3; moderate r < 0.5; strong r > 0.5) [18]. A partial correlation analysis was undertaken to measure the strength of a relationship while controlling for another variable. For multiple pairwise comparisons an adjusted Bonferroni correction was made.

Finally, we performed an item analysis to review the psychometric properties of the mood rating scales and the contributions of *somatic* and *non-somatic* components. The BAI, BDI-II and HADS scales were split into groupings of items that are likely to reflect the influence of motor symptoms and items that were likely not influenced by motor symptoms. The means between these groupings were reviewed. Cronbach’s alpha (a measure of closely related a set of items are as a group) was assessed for internal consistency. Principal component analysis was performed to divide the mood questionnaires into its components and then compared with our *a priori* groupings as shown below.

IBM SPSS 27 was used for analysis. Normality was tested for using Shapiro-Wilks and Kolmogorov-Smirnov tests.

## 3 Results

### 3.1 Participants

201 participants (65 men/ 136 women) with cervical dystonia, attending the weekly BoNT clinic for many years, were included for analysis. Mean age overall was 61.5 years (±13 years). Mean age of onset of CD was 43.8 years (±13 years).

### 3.2 Mood assessment scores (Table 1)

86/201 (42 %) participants had either significant depression/anxiety symptoms, or both, when measured by either BAI/BDI-II or both. Mean BAI and BDI-II scores were 11.1 (±10.9) and 11.4 (±10.3) respectively. 62 patients had a BAI ≥ 13; 74 had BDI-II ≥ 13.

104/201 (51 %) of patients met criteria for significant mood symptoms as measured by the either HADS-A/HADS-D or both. Mean HADS-A was 6.4 (±5.4); mean HADS-D, 7.0 (±4.4). 57 participants scored ≥ 13 on BDI-II and ≥ 8 on HADS-D. 42 participants scored ≥ 13 on BAI and ≥ 8 on HADS-A.

**Table 1:** The percentages of the 201 participants meeting cut-off for HADS-A, HADS-D, BAI and BDI-II.

Participants (total = 201)	HADS-A ≥ 8 (n = 76)	BAI ≥ 13 (n = 62)	HADS-D ≥ 8 (n = 93)	BDI-II ≥ 13 (n = 74)	BAI and/or BDI-II abnormal
Men (n = 65)	21 (32.3 %)	18 (27.7 %)	30 (32.3 %)	18 (27.7 %)	
Women (n = 136)	55 (40.4 %)	44 (32.4 %)	63 (46.3 %)	56 (41.2 %)	
All participants	76/201 (38 %)	62/201 (30.8 %)	93/201 (46.3 %)	74/201 (36.8 %)	86/201 (42 %)
Mean age	58 years	58 years	59 years	59 years	

HADS-A, Hospital Anxiety and Depression Scale- Anxiety; HADS-D, Hospital Anxiety and Depression Scale- Depression; BAI, Beck Anxiety Inventory; BDI-II, Beck Depression Index 2nd edition.

#### 3.2.1 Influence of TWSTRS2-S on BAI/BDI-II

Patients with BAI and/or BDI-II ≥ 13 had a mean TWSTRS2-S of 11; patients with BAI and/or BDI-II < 13 had a mean score of 10.3. There was no significant difference in mean scores between the two groups [1.0 (±0.66), t(199) = 1.536, p = 0.126]. There was a poor correlation between BAI and BDI-II and TWSTRS2-S, [*r* = 0.152 (p = 0.16) and *r* = 0.124 (p = 0.04) respectively]. A Bonferroni adjusted threshold was not met.

A partial correlation was employed to assess this relationship while controlling for age. There was a poor correlation between TWSTRS2-S and BAI > 13 and BDI-II > 13 whilst controlling for age, r(59) = 0.234, p = 0.7 and r(71) = 0.202, p = 0.86.

#### 3.2.2 Influence of TWSTRS2-S on HADS

Patients with HADS-A and/or HADS-D ≥ 8 had a mean TWSTRS2-S of 11; patients with HADS-A and/or HADS-D of < 8 had mean TWSTRS2-S of 9.9. There was no significant difference in mean scores between the two groups [1.7 (±0.6), t(199) = 1.6, p = 0.882]. The HADS-D and HADS-D were poorly correlated with TWSTRS2-S [*r* = 0.14 (p = 0.021) and *r* = 0.163 (p = 0.048) respectively].

Controlling for age, a partial correlation analysis showed a poor correlation between TWSTRS2-S and HADSA ≥ 8, r(73) = 0.211, p = 0.69, and HADSD ≥ 8, r(83) = 0.176, p = 0.11.

#### 3.2.3 Mood scores and CDIP58 subscales (Table 2)

Mean CDIP-58 Total for 201 patients was 32.7 (±20.8). As expected, the “head and neck” subdomain and “pain” were the subscales with the highest scores with means of 52 and 45 respectively. As shown in Table 2, the strongest correlations were for HADS-A and HADS-D ≥ 8 and sleep subdomain. The CDIP58 correlated strongly only in patients with BAI ≥ 13. Strong correlations were not noted for mood symptoms and motor domains (e.g. upper limb, head and neck and walking).

**Table 2:** Pearson’s correlation values (r) are shown for rating scales assessing anxiety and depression and CDIP58 subscales; poor correlation < 0.3; moderate < 0.5; strong > 0.5.

N = 201	BAI ≥ 13	BDI-II ≥ 13	HADS-A ≥ 8	HADS-D ≥ 8
CDIP-58 Total (mean = 32.7)	***r* = 0.532; p= <0.001**	*r* = 0.456; p < 0.001	*r* = 0.491; p < 0.001	*r* = 0.418; p < 0.001
Head and neck (mean = 52)	*r =* 0.336; p = 0.002	*r* = 0.287; p = 0.013	*r* = 0.374; p < 0.001	*r* = 0.260; p = 0.016
Pain (mean = 45)	*r* = 0.401; p < 0.001	*r* = 0.343; p = 0.003	*r* = 0.421; p < 0.001	*r* = 0.226; p = 0.037
Sleep (mean = 26.8)	*r* = 0.358; p < 0.001	*r* = 0.463; p < 0.001	***r* = 0.514; p < 0.001**	***r* = 0.511; p < 0.001**
Upper limb (mean = 31.6)	*r* = 0.385; p < 0.001	*r* = 0.206; p = 0.078	*r* = 0.307; p = 0.007	*r* = 0.301; p = 0.005
Walking (mean = 25.7)	*r* = 0.418; p < 0.001	*r* = 0.267; p = 0.022	*r* = 0.323; p = 0.004	*r* = 0.272; p = 0.011
Annoyance (mean = 28.7)	*r* = 0.429; p < 0.001	*r* = 0.427; p < 0.001	***r* = 0.510; p < 0.001**	*r* = 0.432; p < 0.001
Mood (mean = 24.1)	*r* = 0.438; p < 0.001	*r* = 0.402; p < 0.001	*r* = 0.461; p < 0.001	*r* = 0.370; p < 0.001
Psychosocial (mean = 34.9)	*r* = 0.330; p = 0.002	*r* = 0.369; p = 0.001	*r* = 0.324; p = 0.004	*r* = 0.349; p = 0.001

Strong correlations that reached statistical significance with Bonferroni correction (36 comparisons) are highlighted in bold.

Controlling for age and TWSTRS2-S as a variable in the strongly correlated values (r > 0.5 in Table 2) suggests that age and motor severity had only a small statistically significant impact on correlation coefficients.

#### 3.2.4 Somatic symptom endorsement in the BAI, BDI-II and HADS-Total

Scores on specific responses to questions/ statements from the questionnaires were available for 149/201 participants (48 men/ 101 women; mean age 61.5-years (±11.6)). These participants were representative of the total population of 201 in mood measures- mean BAI was 11.3, BDI-II was 11.7, mean HADS-A was 6.6 and, mean HADS-D was 7.3. In these 149 participants, we selected items within these instruments most likely to represent or relate to motor difficulties.

#### 3.2.5 BDI-II somatic symptoms

For the BDI-II, the questions were divided into *somatic* and *non-somatic* items. This was arbitrary, but performed in a logical fashion and replicated from a previous study in Parkinson’s Disease [19]. Although that study was based on BDI first edition, the questions are similar and the same items were suitable for analysis here. Items 15–21 were selected to represent somatic symptoms from the BDI-II: loss of energy; changes in sleep pattern; irritability; change in appetite; concentration difficulty; tiredness; loss of interest in sex.

#### 3.2.6 BAI somatic symptoms

For the BAI, previous studies had used a much larger number of questions e.g. 14 items in one study [20] to differentiate somatic and non-somatic and three in another involving patients with Parkinson’s Disease [21]. Neither appeared suitable for CD; for our purposes we selected 5 questions/ statements that overlapped with symptoms frequently reported by our patients**-** items 1, 4, 8, 12, 13 (numbness or tingling; unable to relax; unsteady; hands trembling; shaky).

#### 3.2.7 HADS-Total somatic symptoms

Three statements were selected from HADS based on motor symptom correlation. These were statements 1, 11, 13 (I wake early and then sleep badly for the rest of night; I am restless and can’t keep still; I feel as if I have slowed down).

We first compared these *subjective* somatic items to the more *objective* TWSTRS2-severity. There was no linear relationship between the total scores of somatic items for each mood rating scale and TWSTRS2-S.

Psychometric properties of the BAI, BDI and HADS (Table 3).

**Table 3:** Responses to mood disorder questionnaires by 149 patients with cervical dystonia.

Mean response rate to statements in Mood Disorder Questionnaires In 149 patients with cervical dystonia					
BDI-II Item number	Mean response	BAI Item number	Mean response	HADS-Total Item number	Mean response
**1**	0.38	1*	0.52	1*	**1.49**
**2**	0.53	**2**	0.58	**2**	0.8
**3**	0.46	**3**	0.59	**3**	0.93
**4**	0.92	4*	**0.88**	**4**	0.81
**5**	0.38	**5**	0.73	**5**	0.93
**6**	0.38	**6**	0.68	**6**	0.91
**7**	0.55	**7**	0.48	**7**	0.67
**8**	0.48	8*	**0.73**	**8**	0.77
**9**	0.13	**9**	0.37	**9**	0.45
**10**	0.47	**10**	0.80	**10**	1.12
**11**	0.53	**11**	0.31	11*	**1.01**
**12**	0.51	12*	**0.52**	**12**	1.07
**13**	0.53	13*	**0.76**	13*	**1.85**
**14**	0.44	**14**	0.47	**14**	**1.33**
15*	**0.96**	**15**	0.3		
16*	**1**	**16**	0.41		
17*	**0.52**	**17**	0.49		
18*	**0.5**	**18**	0.65		
19*	**0.69**	**19**	0.27		
20*	**0.96**	**20**	0.44		
21*	**0.94**	**21**	0.51		

Mean results for each item on mood assessments. * represents somatic items. BAI = Beck Anxiety Inventory, BDI-II = Beck Depression Index second edition, HADS = Hospital Anxiety And Depression Scale-Total.

Scores on specific responses to questions/ statements from the questionnaires were available for 149 participants (48 men/ 101 women). For this subgroup, the mean BAI was 11.3, mean BDI-II 11.8, mean HADS-anxiety 6.6 and mean HADS-depression 7.3.

We assessed which items within these scales did patients with CD score highest on. For the BAI, BDI-II and HADS, the single item with highest mean response was part of the *somatic* group. In the BDI-II question 15 and 20 (loss of energy and fatigue respectively) had the highest mean score of 0.96 for both. The highest mean score (0.88) in the BAI was for item 4- inability to relax. The highest mean responses for HADS-Total were seen for item 1 (1.49, wake early and sleep badly for the rest of the night), 13(1.85, I feel as if I have slowed down) and 14(1.33, worrying thoughts constantly go through my mind).

Overall, the somatic group had significantly higher mean scores for each mood disorder instrument than the non-somatic group; BDI-II 0.79 vs 0.51; BAI 0.57 vs 0.51; HADS-T 1.45 vs 0.98 (p < 0.05).

### 3.3 Internal consistency coefficients

Next, using a single dimension construct for the three instruments (anxiety, depression and anxiety and/or depression), we measured Cronbach’s α (Cα; values > 0.7 indicates a good level of internal consistency [22]). The BAI had a Cα of 0.94 (very high internal consistency), the BDI-II a Cα of 0.84 and, the HADS of 0.86 (Table 4). Internal consistency coefficients (Cα) were then measured for the BAI, BDI-II and HADS focused on the a priori somatic and non-somatic groupings. Results indicate high level of reliability throughout.

**Table 4:** Internal consistency coefficients (Cα) for BDI-II, BAI and HADS as well as somatic and non-somatic groupings. *BDI-II = Beck Depression Inventory second edition, BAI = Beck Anxiety Index, HADS = Hospital anxiety and depression score, Cα = Cronbach’s alpha.*

Grouping	Cervical dystonia-Cα
BDI-II	0.84
BDI-II – somatic	0.808
BDI-II – non somatic	0.75
BAI	0.94
BAI – somatic	0.801
BAI – non somatic	0.932
HADS	0.86
HADS – somatic	0.505
HADS – non somatic	0.841

### 3.4 Principal component analysis

Finally we performed a principal component analysis (PCA) to see if factor loadings matched our *a priori* somatic and non-somatic groupings. All items had a loading of at least 0.4. Review of eigenvalues (>1) and scree plots revealed 3 components for the HADS accounting for 67 % of variation, 4 components for the BAI explaining 69 % of the variation and 2 components for the BDI-II accounting for 74 % of the variation.

On visual inspection there was no relationship between the groupings on PCA and our groupings of somatic (motor) and non-somatic symptoms. Motor representations (as we had identified them) were not a significant feature in any of the groupings. No simple structure was noted for the identified components for any of the mood instruments.

## 4 Discussion

In this study of 201 cervical dystonia patients we have noted a number of findings:

Motor scores (TWSTRS2-S) and mood scores: (BAI, BDI-II, and HADS) correlated poorly. Similarly, the CDIP-58 “head and neck” domain also did not correlate strongly with the BAI, BDI-II or HADS.

The “Sleep” subdomain showed strong correlation only with the HADS-A/HADS-D. This may be explained by the fact that patients with anxiety and depression often have sleep issues [23], [24]. However, the BDI-II and BAI were not similarly correlated, even though the BDI-II is the only tool that specifically questions sleep symptoms.

Quality of life: Apart from the BAI, none of the other mood assessment measures correlated strongly with CDIP-58 total.

Somatic and non-somatic representations: Our patients scored higher on the selected somatic grouping, indicating a potential stronger influence of these questions. The single highest scoring item on all three mood questionnaires was a somatic item. However, our PCA show did not show any distinct factor groupings, in fact there was no clear structure noted to any of components even though internal consistency was maintained for our constructs.

Cut-off scores for mood scales: The cut-off scores we used for the BAI, BDI-II and HADS scales may be relevant. A higher proportion of our participants met criteria for excess mood symptoms by HADS-A and HADS-D than by BAI and BDI-II. Also, using HADS-D, we noted an equal prevalence among men and women for depressive symptoms, an unusual finding. These observations may relate to the cut-off scores chosen; even though there is no *ideal* cut-off score, the same measures, using similar cut-offs, have been used in one large focal dystonia study [4].

Cervical Dystonia (CD) is characterised by motor heterogeneity (variations in dystonic posturing) and non-motor features (psychiatric, cognitive and sensory) [25]. It has been suggested that CD can be clinically divided into two clusters with similar motor features and non-motor features of varying severity with age acting as a modulating factor [26]. Some authors have proposed that motor severity and psychiatric symptoms are directly related [27]. One study demonstrated a reduction in MADRS (Montgomery and Asberg Depression Rating Scale) scores from BoNT A treatment and improvement in all aspects of the SF-36 [28]. The role of BoNT is particularly interesting as it has been suggested that it can have an antidepressant effect across a variety of disorders [29], [30]. However in CD, most evidence is against mood symptoms being purely reactive to motor disability- mood and motor symptoms have no correlation [26], improvement in motor features does not result in improvement in anxiety or depression [31] and, patients with CD shower higher rates of psychiatric comorbidity when compared to another cosmetically disfiguring disorder [32].

Proposed pathophysiological models must incorporate both the motor and non-motor symptoms. Serotonergic pathways [33] are key in mediating dystonia onset; specifically, serotonin transporter binding has been shown to correlate with the motor and non-motor features [34]. Anatomically, we have proposed that a network involving superior colliculus, pulvinar nucleus and amygdala could explain the entire syndrome of CD [35]. Cells enriched for heritability of psychiatric disorders are also enriched for known dystonia associated genes. We hypothesise that further research determining the presence of mood disorder in apparently unaffected relatives of patients with cervical dystonia could lead to discovery of new gene variants.

This study has limitations. CD is a rare, likely underdiagnosed, disorder which can make recruitment of large populations difficult [36]. Ours is one of the larger studies on this topic, but phenotypic differences affecting a large number of muscles [37] can make it difficult to identify homogenous populations within CD.

We would suggest that clinicians be mindful that symptoms of a mood disorder can be present even in patients with mild motor symptoms. For an holistic assessment, one should employ measures of QoL, motor severity and mood disorder. Neuropsychology integration as part of a dystonia service could greatly enhance patient outcomes. Use of rating scales are particularly important where neuropsychology resources are limited. Patients could be screened routinely with high scoring patients receiving referral for psychology input. This approach could obviate the need for pharmacotherapy.

A useful future study would be to design a brief holistic assessment instrument to provide a reliable measure of the multiple facets of the syndrome of cervical dystonia. This would require international collaboration.

Informed consent was obtained from all participants

We confirm that we have read the Journal’s position on issues involved in ethical publication and affirm that this work is consistent with those guidelines.”

SR: no relevant financial disclosures or conflicts of interest.

MAH: no relevant financial disclosures or conflicts of interest.

GD: no relevant financial disclosures or conflicts of interest.

IN: no relevant financial disclosures or conflicts of interest.

MH: no relevant financial disclosures or conflicts of interest.

## 5 Author roles

(1) Project: A. Conception, B. Organization, C. Execution; (3) Manuscript: A. Writing of the First Draft, B. Review and Critique.

SR: 1A, 1B, 1C, 3A

MAH: 1A, 1B, 1C, 3A

GD: 1B, 1C

ID: 1A, 1B, 1C

MH: 1A, 1C, 3A, 3B

## CRediT authorship contribution statement

**Shameer Rafee:** Conceptualization, Methodology, Writing – review & editing. **Mahmood Al-Hinai:** Data curation, Writing – original draft. **Gillian Douglas:** Data curation. **Ihedinachi Ndukwe:** Methodology. **Michael Hutchinson:** Conceptualization, Writing – review & editing.

## Declaration of Competing Interest

The manuscript was supported by funding from Dystonia Ireland.
